# The HIV/AIDS Epidemiological Challenges: Why is HIV Incidence Rising While AIDS Incidence and Mortality are Declining in Brazil?

**DOI:** 10.1007/s10900-026-01590-x

**Published:** 2026-06-18

**Authors:** Henrique Fernando Lopes-Araujo, Maria Carolina Santos Guedes, Wlisses Henrique Veloso Carvalho-Silva, Rafael Lima Guimarães

**Affiliations:** 1 Department of Genetics, Federal University of Pernambuco – UFPE, Recife, PE 50670-901 Brazil; 2 Keizo Asami Institute (iLIKA), Federal University of Pernambuco – UFPE, Recife, PE 50670-901 Brazil; 3 Department of Immunology, Aggeu Magalhães Institute (IAM) – Oswaldo Cruz Foundation (FIOCRUZ), Recife, PE 50740-465 Brazil; 4 FACOL University Center - UNIFACOL, Vitória de Santo Antão, Pernambuco 55612-285 Brazil

**Keywords:** Antiretroviral therapy, Public health, Treatment initiation, Viral suppression

## Abstract

HIV/AIDS remains a public health challenge in Latin America, and Brazil has emerged as a global reference for effective epidemiological control through its comprehensive public health response. This study investigated epidemiological trends in Brazil from 2015 to 2024, including HIV and AIDS incidence, AIDS-related mortality, time from diagnosis to antiretroviral therapy initiation, CD4 + T cell counts at diagnosis, and viral suppression. We aimed to evaluate progress in HIV/AIDS management, identify persistent gaps requiring improvement, and explore potential determinants associated with AIDS incidence and mortality to inform future policy and programmatic actions. During this period, AIDS incidence and AIDS-related death rates declined, but the HIV incidence rate increased. The percentage of people living with HIV initiating treatment within 30 days after diagnosis increased from 28 to 63%, while the proportion initiating treatment more than 6 months after diagnosis decreased substantially from 35 to 10%. Viral suppression among people living with HIV and CD4 + T cell counts at diagnosis remained relatively stable. Moreover, we observed a strong negative correlation between treatment initiation within 30 days and the AIDS incidence rate (r = -0.8147; p = 0.0075), as well as a very strong negative correlation with the AIDS-related death rate (r = -0.9564; p < 0.0001). Therefore, Brazil reduced AIDS incidence and mortality through earlier treatment initiation and sustained viral suppression. However, persistently high HIV incidence remains a challenge, requiring intensified prevention campaigns, stigma reduction, and targeted interventions in high-risk populations.

**Supplementary Information:**

The online version contains supplementary material available at https://doi.org/10.1007/s10900-026-01590-x.

## Introduction

The human immunodeficiency virus (HIV) infects immune cells through binding to the CD4 receptor and to the CCR5 and/or CXCR4 coreceptors on the cell surface [6, 24]. In the absence of treatment, HIV infection is characterized by persistent viremia and a progressive decline in CD4 + T cell counts [11, 45]. The final stage of the untreated infection is a profound immunodepression, typically indicated by CD4 + T cell counts below 200 cells/µL, and is clinically defined as acquired immunodeficiency syndrome (AIDS) [23, 28]. During this stage, people living with HIV (PLHIV) exhibit a high risk of morbidity due to opportunistic infections, HIV-associated malignancies, and other AIDS-related complications [20, 22].

Antiretroviral therapy (ART) was introduced in the 1990s and has since evolved into a highly effective intervention that has transformed HIV infection into a manageable chronic condition, whose impact has been monitored through epidemiological analyses over the years. ART suppresses viral replication and reduces plasma viral loads to undetectable and untransmissible levels (< 50 copies of viral RNA/mL) [40, 47]. Current guidelines for the anti-HIV treatment recommend the use of multiple drug classes, targeting distinct mechanisms to inhibit the viral replication cycle, optimize viral suppression and reduce the emergence of drug resistance [48]. Effective ART contributes to immune recovery by suppressing viral load, limiting CD4 + T cell depletion, and reducing the risk of progression to AIDS [8, 19, 38]. Moreover, sustained viral suppression reduces the incidence of HIV-related comorbidities that compromise the quality of life and survival of PLHIV, including tuberculosis and non-Hodgkin lymphoma, which exhibits an increased incidence in individuals with uncontrolled viremia [24, 26, 33, 34].

The Joint United Nations Programme on HIV/AIDS (UNAIDS) estimated that more than 88 million people have been infected with HIV worldwide since the beginning of the epidemic, and that over 42 million AIDS-related deaths occurred during this period [42]. Substantial progress has been made in both the prevention and treatment of HIV infection over the past four decades since the emergence of the HIV epidemic [32]. Despite these advances, HIV/AIDS remains a major global public health challenge, particularly in low and middle-income countries such as Brazil [7]. Data indicate that, since the beginning of the epidemic, more than 1.6 million people living with HIV have been reported in the country, with approximately 400,000 deaths estimated to have HIV/AIDS recorded as the underlying cause [4]. Although Brazil stands out as a global model in the management of the HIV/AIDS pandemic due to its comprehensive public health response, HIV infection and AIDS remain significant health concerns in the country [17]. The persistence of these challenges is associated with social and regional inequalities in the country, as well as delayed diagnosis and barriers to sustained engagement in HIV care.

Therefore, this study sought to analyze epidemiological trends in Brazil over the past decade (2015–2024), focusing on relative metrics. This analysis aims to elucidate trends and advances in the management of HIV/AIDS in the country, while also evaluating and identifying gaps that require further improvement, with the aim of contributing to the strengthening of monitoring strategies and the management of PLHIV. Moreover, we explored potential factors that could be correlated with AIDS incidence and AIDS-related mortality.

## Materials and Methods

### Study Design and Data Collection

This study employed a retrospective ecological time-series design, analyzing data from 2015 to 2024. Data regarding HIV incidence, AIDS incidence, AIDS-related deaths as the primary cause, including absolute case counts and detection rates per 100,000 inhabitants, were obtained from the Brazilian Epidemiological Reports on HIV and AIDS (available at https://www.gov.br/aids/pt-br/central-de-conteudo/boletins-epidemiologicos; accessed December 13, 2025). Absolute counts and detection rates were analyzed using Microsoft Excel (version 2018), with detection rates serving as the primary relative metric for epidemiological analyses and correlation tests. Meanwhile, data regarding viral load among PLHIV, the time from diagnosis to ART initiation, and the CD4 + T cell count at diagnosis were retrieved from the Integrated Monitoring Panel for HIV and AIDS Care (available at: https://www.gov.br/aids/pt-br/indicadores-epidemiologicos/painel-de-monitoramento; accessed December 13, 2025). Absolute counts were extracted from the panel and analyzed using Microsoft Excel (version 2018), where percentages within stratified groups were calculated and used as the primary relative metric for epidemiological analyses and correlation tests. All data were sourced exclusively from official public databases of the Brazilian Ministry of Health, thereby eliminating risks to individual privacy and ensuring adherence to ethical standards.

### Statistical Analyses

For numerical variables, Gaussian distribution was evaluated using the Shapiro–Wilk test. Variables were displayed as relative and absolute frequencies. Pearson’s correlation test was employed for normally distributed data, while Spearman’s correlation test was used for non-normally distributed data. Correlation’s strengths were classified as follows: none (r = 0.00), very weak (r = 0.01 to 0.19), weak (r = 0.20 to 0.39), moderate (r = 0.40 to 0.69), strong (r = 0.70 to 0.89), very strong (r = 0.90 to 0.99), and perfect (r = 1), adapted from Schober et al*.* [36]. The statistical significance level (α) was set at 0.05 for all tests, with a 95% confidence interval. Statistical analyses and graphical representations were performed using GraphPad Prism (version 8.0).

## Results

We analyzed trends in major HIV/AIDS indicators from 2015 to 2024, including HIV incidence, AIDS incidence, and AIDS-related death detection rate per 100,000 inhabitants. We also analyzed the percentages of undetectable viral load among PLHIV, time from HIV diagnosis to treatment, as well as baseline CD4 + T cell count at diagnosis (Table 1).

**Table 1:** HIV/AIDS indicators in Brazil from 2015 to 2024

	2015	2016	2017	2018	2019	2020	2021	2022	2023	2024
*Detection rate (per 100.000)*										
HIV incidence	18.0	18.2	19.0	19.5	18.9	15.8	17.5	17.2	18.1	18.4
AIDS incidence	19.3	18.7	18.3	18.3	18.1	14.3	16.7	17.3	17.7	17.4
AIDS related-death	5.3	5.2	4.8	4.5	4.2	4.1	4.4	4.2	3.9	3.4
*Viral load of ART-treated PLHIV*										
Detectable (%)	17.87	16.37	15.47	12.60	11.89	10.04	9.93	10.11	11.97	14.06
Undetectable (%)	82.13	83.63	84.53	87.40	88.11	89.96	90.07	89.89	88.03	85.94
*Time from diagnosis to ART*										
< 30 days (%)	28.12	32.48	35.15	40.67	42.57	48.69	49.66	51.13	57.34	62.71
1–6 months (%)	37.21	42.21	42.78	41.84	41.35	37.33	36.09	36.53	32.21	27.66
> 6 months (%)	34.67	25.31	22.07	17.49	16.08	13.98	14.25	12.34	10.45	9.63
*CD4 + T cell count at diagnosis*										
≥ 200 (%)	73.41	73.73	73.92	73.99	73.73	73.23	73.81	72.48	73.48	74.72
< 200 (%)	26.59	26.27	26.08	26.01	26.27	26.77	26.19	27.52	26.52	25.28

ART: antiretroviral therapy; PLHIV: people living with HIV

**Fig. 1:**
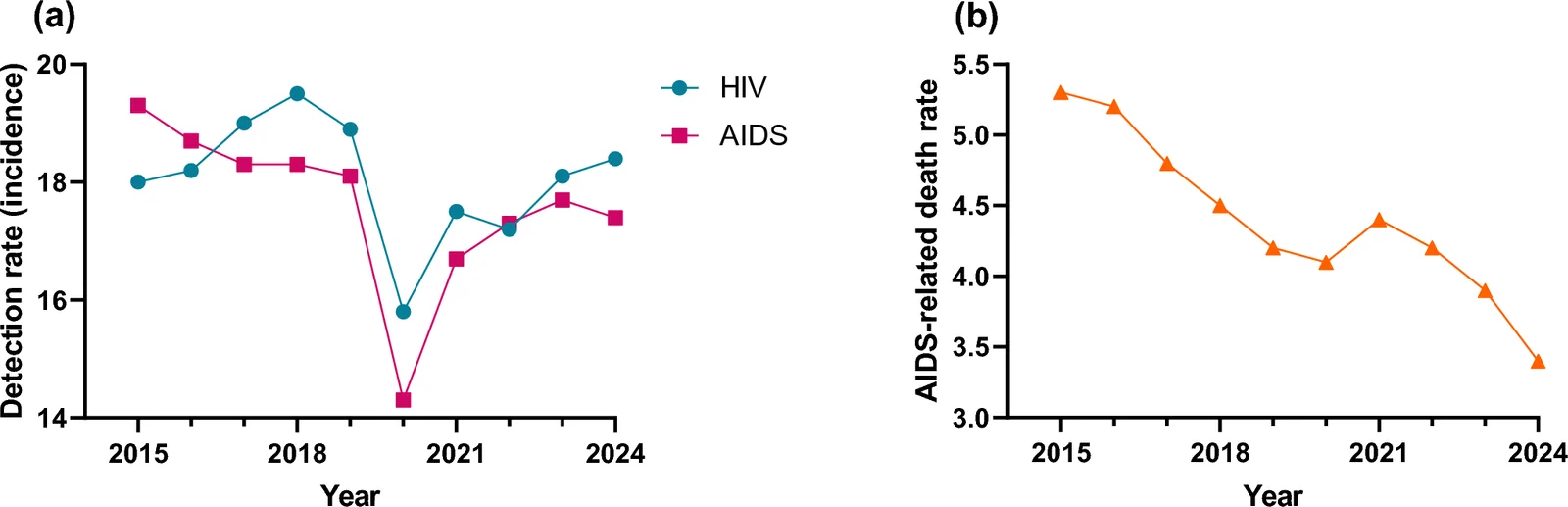
HIV/AIDS incidence and mortality in Brazil from 2015 to 2024. (**A**) HIV and AIDS incidence rates. (**B**) AIDS-related death rates

Between 2015 and 2024, the HIV detection rate per 100,000 inhabitants increased from 18.0 to a peak of 19.5 cases in 2018, before declining and then stabilizing at 18.4 in 2024. During the same period, AIDS detection rate per 100,000 inhabitants demonstrated an overall decline, decreasing from 19.3 in 2015 to 17.4 in 2024. There was a sharp decline in both HIV and AIDS incidence in 2020, likely influenced by underreporting due to the COVID-19 pandemic (Fig. 1A). Meanwhile, the AIDS-related death rate per 100,000 inhabitants followed a steady downward trend, falling from 5.3 to 3.4 deaths between 2015 and 2024 (Fig. 1B).

**Fig. 2:**
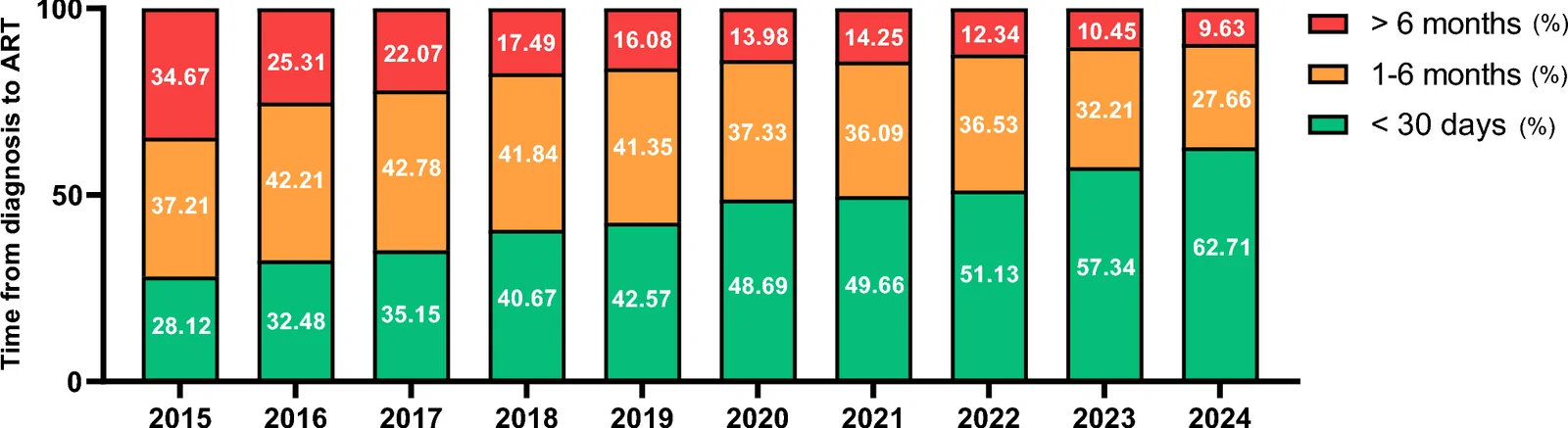
Annual distribution and proportion of time from HIV diagnosis to ART initiation among PLHIV from 2015 to 2024 in Brazil

During the period from 2015 to 2024, the percentage of PLHIV initiating ART within 30 days after HIV diagnosis increased from 28.12% to 62.71%. As a consequence, the percentage of PLHIV initiating ART after more than six months decreased progressively from 34.66% to 9.63% (Fig. 2).

**Fig. 3:**
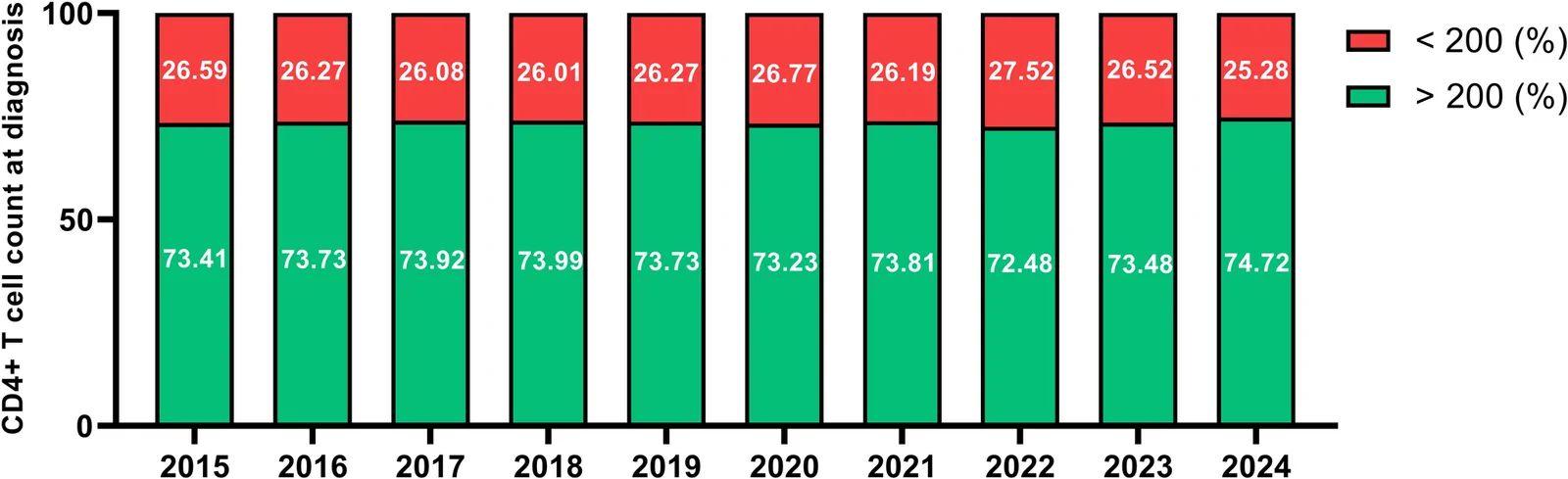
Annual distribution and proportion of CD4 + T cell counts at diagnosis from 2015 to 2024 in Brazil

Between 2015 and 2024, the percentage of PLHIV initiating treatment with baseline CD4 + T cell count below 200 cells/µL remained stable, ranging from 25.28% to 27.52%, whereas the proportion of individuals starting treatment with count above 200 cells/µL was also stable, fluctuating between 72.48% and 74.72% (Fig. 3). These results demonstrate limited year-to-year variations over the period for CD4 + T cell counts at diagnosis.

**Fig. 4:**
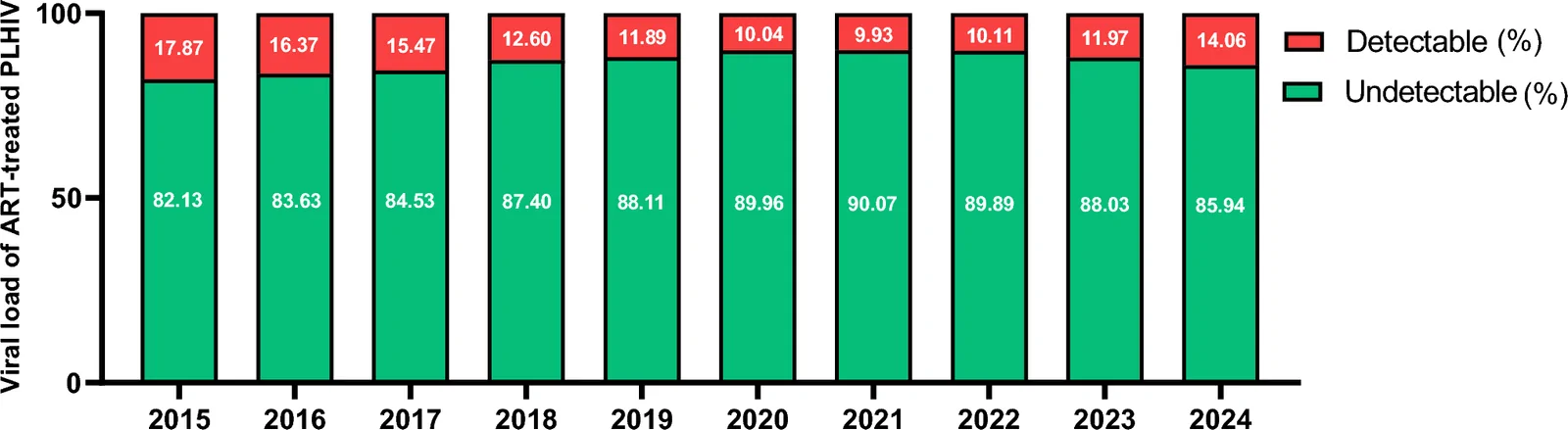
Annual distribution and proportion of viral loads among ART-treated PLHIV in Brazil from 2015 to 2024

The percentage of ART-treated PLHIV with undetectable viral load remained stable from 2015 to 2024, ranging from 82.13% in 2015 to a peak of 90.07% in 2021, before returning to 85.94% in 2024. Accordingly, the percentage of ART-treated PLHIV with detectable viral load decreased from 17.87% in 2015 to 9.93% in 2021, before increasing to 14.06% by 2024 (Fig. 4). Overall, these changes reflect relative stability in viral suppression among PLHIV under ART, with limited year-to-year variations over the period.

**Fig. 5:**
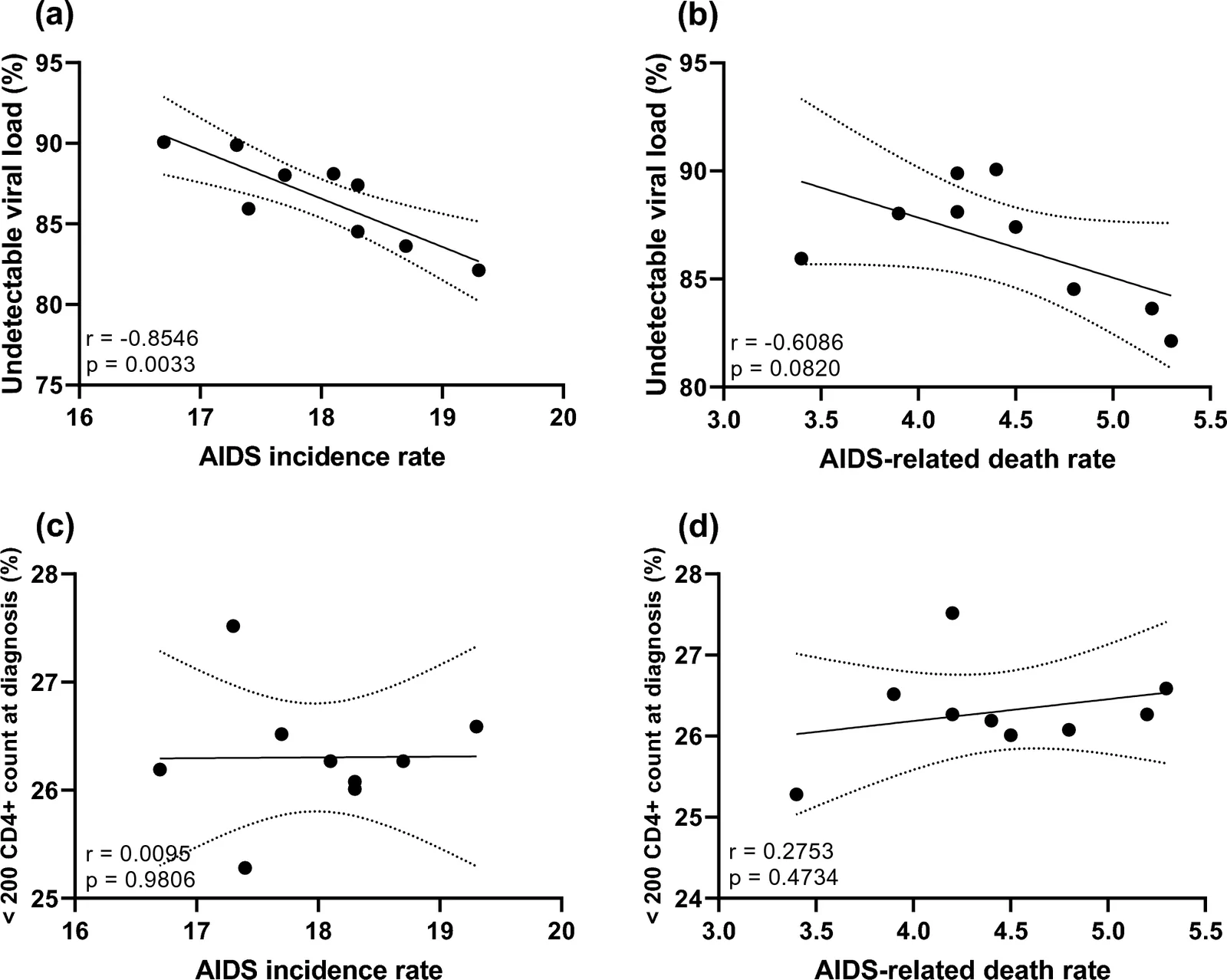
Correlations between AIDS epidemiological rates and viral load and CD4 + T cell count at diagnosis in Brazil from 2015 to 2024. (**A**) AIDS incidence rate and undetectable viral load. (**B**) AIDS incidence rate and < 200 CD4 + T cell count at diagnosis. (**C**) AIDS-related death rate and undetectable viral load. (**D**) AIDS-related death rate and < 200 CD4 + T cell count at diagnosis

Correlation analyses revealed a strong, negative, and statistically significant correlation between the percentage of PLHIV with undetectable viral load and the AIDS incidence rate over the years (r = -0.8546; p = 0.0033; Fig. 5A). No statistically significant correlation was observed between the AIDS incidence rate and the percentage of individuals initiating ART with baseline CD4 + T cell count below 200 cells/µL (r = 0.0095; p = 0.9806; Fig. 5B). Additionally, there was no statistically significant correlation of AIDS-related mortality rate with the percentage of individuals with viral suppression (r = -0.6086; p = 0.0820; Fig. 5C), nor with percentage of individuals initiating ART with baseline CD4 + T cell count below 200 cells/µL (r = 0.2753; p = 0.4734; Fig. 5D).

**Fig. 6:**
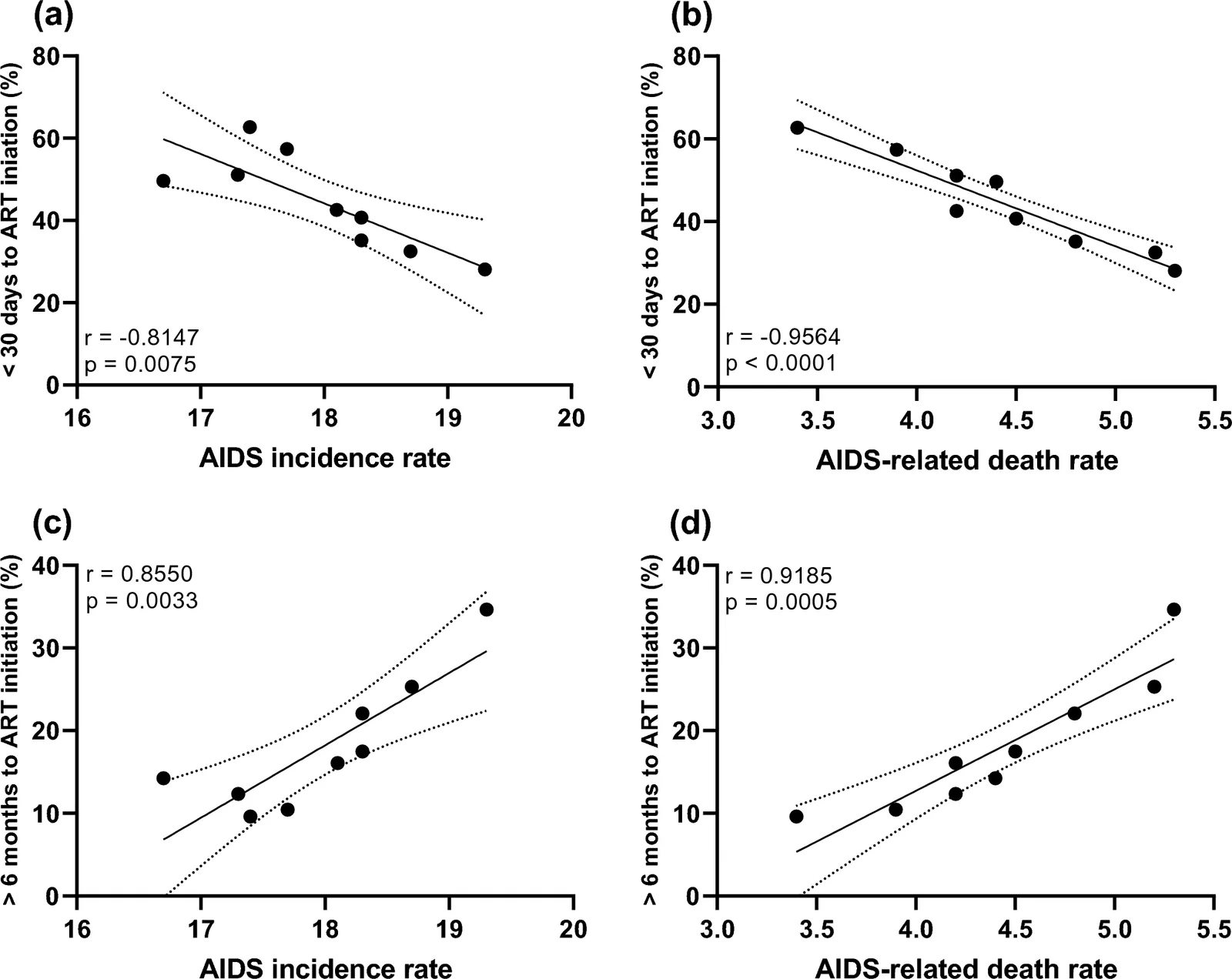
Correlations between AIDS epidemiological rates and time from HIV diagnosis to ART initiation in Brazil from 2015 to 2024. (**A**) AIDS incidence rate and < 30 days to ART initiation. (**B**) AIDS-related death rate and < 30 days to ART initiation. (**C**) AIDS incidence rate and > 6 months do ART initiation. (**D**) AIDS-related death rate and > 6 months to ART initiation

A strong, negative and statistically significant correlation was also observed between the percentage of PLHIV who initiated ART within 30 days after diagnosis and the AIDS incidence rate over time (r = -0.8147; p = 0.0075; Fig. 6A). Moreover, between early treatment initiation (< 30 days) and AIDS-related death rate, the correlation was very strong, also negative and statistically significant (r = -0.9564; p < 0.0001; Fig. 6B). Conversely, the percentage of individuals initiating treatment more than six months after diagnosis demonstrated a strong, positive and statistically significant correlation with the AIDS incidence rate (r = 0.8550; p = 0.0033; Fig. 6C), as well as a very strong, positive and statistically significant correlation with the AIDS-related death rate (r = 0.9185; p = 0.0005; Fig. 6D).

The correlation tests excluded 2020, as this year exhibited outliers that biased the results. All analyses and figures were based on relative measures, including rates per 100,000 inhabitants and percentages, to facilitate appropriate temporal comparisons, accounting for changes in population dynamics. This approach facilitates the interpretation of trends over time and minimizes the influence of variations in population size in epidemiological studies. Moreover, absolute counts for all variables analyzed in our study across the years (2015 to 2024) are provided in the supplementary material (Supplementary Table S1).

## Discussion

Over the past 40 years, ART has transformed HIV infection from a fatal illness into a manageable chronic condition, enabling ART-treated PLHIV to achieve near-normal life expectancy and good quality of life [15]. Nevertheless, HIV/AIDS continues to pose a significant public health challenge worldwide, including in Brazil and across Latin America, where persistent gaps in prevention, diagnosis, and care demand ongoing research to optimize clinical management and outcomes [12]. Our results revealed a decline in both AIDS incidence and the AIDS-related death rate between 2015 and 2024. In contrast, HIV incidence did not decrease and, in fact, indicated an upward trend over the period. The persistent increase in HIV incidence observed in this study may reflect multiple factors, including changes in sexual behavior, inconsistent condom use, disparities in access to preventive services, and increased HIV testing coverage, which may lead to greater detection of previously undiagnosed infections. Notably, in 2015, the AIDS incidence rate exceeded that of HIV infection; this pattern reversed over time, such that by 2024, the HIV incidence rate surpassed the AIDS incidence rate. The reduction of AIDS incidence, despite rising HIV infections, may be attributed to effective public health management strategies. It is also worth noting a sharp drop in reported HIV/AIDS incidence in 2020, most probably due to underreporting during the COVID-19 pandemic, a phenomenon observed in several other countries during the pandemic [13, 37, 49].

Early ART initiation is a critical factor in reducing mortality risks while improving the quality of life among PLHIV [29, 38]. Our results align with this evidence by demonstrating statistically significant negative correlations between the percentage of individuals starting treatment within 30 days of diagnosis and the AIDS incidence rate, as well as the AIDS-related death rate. However, these correlations should be interpreted cautiously, as multiple structural, behavioral, and healthcare-related factors may also influence HIV epidemiological trends. The World Health Organization advocates a “test-and-treat” approach, recommending immediate ART initiation upon HIV diagnosis regardless CD4 + T cell count [46]. Evidence indicates that such early ART not only promotes viral suppression but also contributes to better CD4 + T cell reconstitution [1, 21]. Moreover, our data demonstrated an improvement in the interval between HIV diagnosis and ART initiation from 2015 to 2024 in Brazil. Whereas half of the diagnosed individuals used to wait over a month to begin anti-HIV treatment, nowadays the majority start within 30 days. Brazil’s policy of providing free, universal access to ART since 1996 has likely contributed to this progress [30, 44]. Nevertheless, PLHIV still experience delays of several months before initiating ART in some cases, thereby elevating their risk of adverse outcomes. It is crucial to understand the factors that lead to late ART initiation: are they related to fear and stigma surrounding diagnosis, barriers to healthcare access, or limited knowledge about the U = U principle?

U = U stands for the “undetectable equals untransmittable” campaign, which means that although ART does not fully eradicate HIV due to persistent viral reservoirs, it can suppress plasma viral load to undetectable levels by standard assays, rendering sexual transmission highly unlikely [2, 18]. Therefore, increasing the percentage of PLHIV who are virally suppressed represents a central strategy for controlling the HIV/AIDS pandemic. Individuals with undetectable viral loads are also far less likely to progress to AIDS, as minimal viral replication limits depletion of immune cells [5]. Our data contributes to this evidence because we also demonstrated a statistically significant negative correlation between the percentage of PLHIV with undetectable viral loads and the AIDS incidence rate. Moreover, limiting viral replication reduces CD4 + T cell depletion and allows the reconstitution of these cells through different mechanisms, for example, the redistribution of memory CD4 + T cells from lymphoid tissues to peripheral blood [9, 19]. The percentage of PLHIV with undetectable viral loads demonstrated limited year-to-year variation over the study period and current proportions indicate that nearly 90% of ART-treated PLHIV are virologically suppressed. Therefore, Brazil is moving toward internationally recognized standards and aligning with the UNAIDS 95–95-95 goals (95% of ART-treated PLHIV must have an undetectable viral load by 2030) [43]. Further improvements remain possible, particularly by addressing persistent barriers to ART initiation and treatment follow-up. Factors such as stigma, delayed treatment uptake, and loss to follow-up (LFU) continue to influence virological outcomes [10, 16]. The LFU arises from diverse factors, including ART side effects, discrimination, absence of family support, and illicit drug use [3, 35]. Certain populations exhibit higher LFU rates: rural residents, those with low educational attainment, and socioeconomic status, where evidence associates food insecurity with lower ART adherence [27, 31, 35].

CD4 + T cell count at HIV diagnosis is important for clinical management. Evidence indicates that individuals who initiate ART with higher CD4 + T cell counts are more likely to achieve sustained viral suppression and adequate immune reconstitution [25, 39]. Despite this well-established importance, our data indicated no significant decline in the percentage of PLHIV diagnosed with low CD4 + T cell counts. Currently, approximately 25% of new diagnoses occur at CD4 + T cell counts below 200 cells/μL, which represents the AIDS stage and is associated with an elevated mortality risk. Late HIV diagnosis accompanied by immunocompromise and low CD4 + T cell count, could be partly explained by the nonspecific clinical manifestations that characterize early HIV infection [14]. Therefore, public awareness campaigns are essential to promote prevention, facilitate recognition of potential signs and symptoms, and emphasize the value of regular testing alongside messages affirming the efficacy of modern anti-HIV treatment. Efforts to encourage appropriate use of self-testing kits are equally important, as Toledo [41] demonstrated that individuals frequently commit errors during self-testing, potentially compromising test accuracy and individual safety [41].

A notable limitation of this study arises from its dependence on publicly available secondary datasets. Additionally, as an ecological study based on aggregated population-level data, the present analysis does not allow causal inferences at the individual level. Although these sources provide valuable breadth and accessibility, they do not allow for the extraction of key granular information, particularly post-diagnosis CD4 + cell counts among ART-treated PLHIV. This gap restricts a more precise evaluation immunosuppression severity, clinical staging, disease progression risk, and treatment strategies for PLHIV, especially those diagnosed late or with suboptimal virologic control.

## Conclusion

These findings reinforce the importance of strengthening integrated HIV control strategies that combine early diagnosis, rapid ART initiation, sustained treatment adherence, and expanded prevention initiatives targeting populations at higher risk of infection. Brazil demonstrated divergent trends in HIV and AIDS epidemiology from 2015 to 2024: a steady decline in the AIDS incidence rate and the AIDS-related death rate alongside a persistent rise in the HIV incidence rate. One important advancement in HIV/AIDS management was the increase in PLHIV initiating ART within one month after HIV diagnosis. Despite these advancements, there are still important gaps that require improvement, for example: high HIV incidence continues to coexist with a substantial proportion of individuals diagnosed with advanced immunosuppression. To further consolidate and improve these achievements regarding HIV/AIDS, the Brazilian government should continue strengthening prevention strategies, particularly evidence-based campaigns to promote the use of condoms and pre-exposure prophylaxis (PrEP). Moreover, campaigns to encourage regular testing and appropriate use of self-testing, and reduce stigma associated with HIV infection and diagnosis are extremely necessary. Additionally, public health strategies should prioritize vulnerable populations at higher risk of late diagnosis or loss to follow-up. Thus, the analysis of HIV epidemiological patterns in the country is essential for monitoring the response to the epidemic and advancing health planning, providing support for progress in the clinical management of AIDS and the reduction of HIV incidence in Brazil.

## Supplementary Information

Below is the link to the electronic supplementary material.

Supplementary file1
