# Supplementary material for: The HIV/AIDS Epidemiological Challenges: Why is HIV Incidence Rising While AIDS Incidence and Mortality are Declining in Brazil?
Source: J Community Health. 2026 Jun 18;51(5):785–94. doi: 10.1007/s10900-026-01590-x (PMC13614994; doi:10.1007/s10900-026-01590-x)
Supplement: Supplementary file 1 [file 10900_2026_1590_MOESM1_ESM.docx]

**Supplementary Table S1**. Absolute numbers of HIV/AIDS indicators in Brazil from 2015 to 2024.

|  | **2015** | **2016** | **2017** | **2018** | **2019** | **2020** | **2021** | **2022** | **2023** | **2024** |
| --- | --- | --- | --- | --- | --- | --- | --- | --- | --- | --- |
| **Incidence** |  |  |  |  |  |  |  |  |  |  |
| HIV incidence | 36.375 | 37.018 | 39.085 | 40.259 | 39.242 | 32.949 | 36.857 | 36.277 | 38.222 | 39.216 |
| AIDS incidence | 39.088 | 38.068 | 37.561 | 37.738 | 37.573 | 29.980 | 35.085 | 36550 | 37527 | 36.955 |
| AIDS related-death | 12.605 | 12.470 | 11.663 | 11.176 | 10.634 | 10.596 | 11.515 | 11.062 | 10.500 | 9.157 |
| **Viral load of ART-treated PLHIV** |  |  |  |  |  |  |  |  |  |  |
| Detectable | 57.502 | 59.787 | 60.313 | 55.261 | 56.568 | 45.004 | 50.496 | 55.334 | 71.530 | 90.380 |
| Undetectable | 264.331 | 305.431 | 329.492 | 383.277 | 418.998 | 403.401 | 457.903 | 491.785 | 525.967 | 552.215 |
| **Time from diagnosis to ART** |  |  |  |  |  |  |  |  |  |  |
| < 30 days | 20.059 | 21.530 | 24.262 | 27.526 | 28.712 | 26.527 | 30.324 | 31.486 | 37.109 | 40.844 |
| 1-6 months | 26.542 | 27.972 | 29.533 | 28.311 | 27.883 | 20.338 | 22.036 | 22.492 | 20.840 | 18.015 |
| > 6 months | 24.725 | 16.773 | 15.237 | 11.840 | 10.843 | 7.619 | 8.705 | 7.600 | 6.765 | 6.270 |
| **CD4+ T cell count at diagnosis** |  |  |  |  |  |  |  |  |  |  |
| ≥ 200 | 32.061 | 33.209 | 34.687 | 36.551 | 36.033 | 28.319 | 33.821 | 34.375 | 37.669 | 39.531 |
| < 200 | 11.610 | 11.832 | 12.237 | 12.847 | 12.841 | 10.354 | 11.999 | 13.050 | 13.598 | 13.371 |
